# Editorial: New advances in the renal regulation of K^+^ homeostasis in health and disease

**DOI:** 10.3389/fphys.2023.1288898

**Published:** 2023-10-12

**Authors:** Gilles Crambert, Lama Al-Qusairi

**Affiliations:** ^1^ Laboratoire de Physiologie Rénale et Tubulopathies, Centre de Recherche des Cordeliers, L’Institut National de la Santé et de la Recherche Médicale, Sorbonne Université, Université Paris Cité, Paris, France; ^2^ CNRS EMR 8228—Unité Métabolisme et Physiologie Rénale, Paris, France; ^3^ Division of Nephrology, Johns Hopkins Medicine, Baltimore, MD, United States

**Keywords:** K^+^ homeostasis, hypokalemia, hyperkalemia, kidney, pathophysiology

Plasma K^+^ level is tightly maintained within a narrow range (3.5–5.0 mM) by complex physiological mechanisms involving coordination of K^+^ storage occurring mainly in skeletal muscles and K^+^ excretion via the kidneys and, to a lesser extent, the colon. These processes are crucial for the proper function of excitable and non-excitable cells. Keeping plasma K^+^ within such a narrow range is a constant challenge because the extracellular [K^+^] can be easily disturbed either by potassium intake as daily intake can be equal to the overall amount of extracellular [K^+^], or by intra-to-extracellular K^+^ shift given the intracellular [K^+^] is much higher than the extracellular [K^+^]. Hence, dyskalemia is common and occurs in many conditions [for review, see (Tabibzadeh and Crambert, 2023)] resulting in adverse clinical outcomes (Palaka et al., 2020).

A recent population study revealed a rising trend of hypokalemia in the US general population from 3% in 1999 to 11% in 2016, likely due to dietary potassium (K^+^) deficiency (Sun and Weaver, 2021). Mild dietary K^+^ deficiency is associated with salt-sensitive hypertension, and severe forms of hypokalemia lead to heart arrhythmias, paralysis, and nephrogenic diabetes insipidus [for review, see (Kardalas et al., 2018)]. Moreover, hypokalemia is a risk factor for chronic kidney disease (CKD) progression. Despite extensive literature exploring several areas of the physiopathology of hypokalemia-associated disorders, the molecular underpinning of K^+^ retention and the associated pathophysiologic cascades are still not fully understood. In a short review, Lasaad and Crambert recapitulated the mechanisms of K^+^ retention in the distal nephron, namely, the distal convoluted tubule (DCT) and the collecting duct (CD). They explained how these mechanisms are linked to the Na^+^ transport system and the regulation of the extracellular compartment volume and blood pressure. Of note, they gave examples of studies where the lack of a K^+^ retention system, like in H,K-ATPase type 2 knock-out mice, under physiological circumstances (K^+^ restriction or gestation) is compensated by inhibition of Na^+^ reabsorption either in the DCT or the cortical CD, inducing a loss of fluid and salt, leading to a reduction of plasma volume. This may contribute to “concentrating” K^+^ in the extracellular compartment and reduce the risk of developing hypokalemia at the detriment of the blood pressure.

Conversely, hyperkalemia is also increasing in the population, particularly CKD patients (Kashihara et al., 2019). The development of hyperkalemia can be explained by the fact that some CKD patients maintain their K^+^ balance (equilibrium between K^+^ input and output) to the detriment of the plasma K^+^ level. In CKD patients, hyperkalemia is correlated to a higher mortality rate by cardiovascular events, and its occurrence is a cause of down-titration or discontinuation of the (Renin-Angiotensin-Aldosterone System inhibitors) RAASi medications with harmful consequences on disease progression. Therefore, optimizing the strategies of hyperkalemia management to control plasma K^+^ in such patients is of primary importance. In this Research Topic, Kanda et al. investigated the clinical outcomes of temporary versus long-term management of hyperkalemia in a nationwide retrospective clinical analysis including more than 4,000 Japanese patients with hyperkalemia. Their study demonstrated that chronic long-term treatment (∼38 months) with potassium binders was more effective than short-term treatment (∼4 months) in reducing hyperkalemia and its associated clinical outcomes, including recurrent hyperkalemia, cardiac events, and hospitalization. Additionally, they found long-term treatment with potassium binder was better than short-term regarding its protective effect against the decline of renal function and was associated with reduced introduction to renal replacement therapy.

**FIGURE 1:**
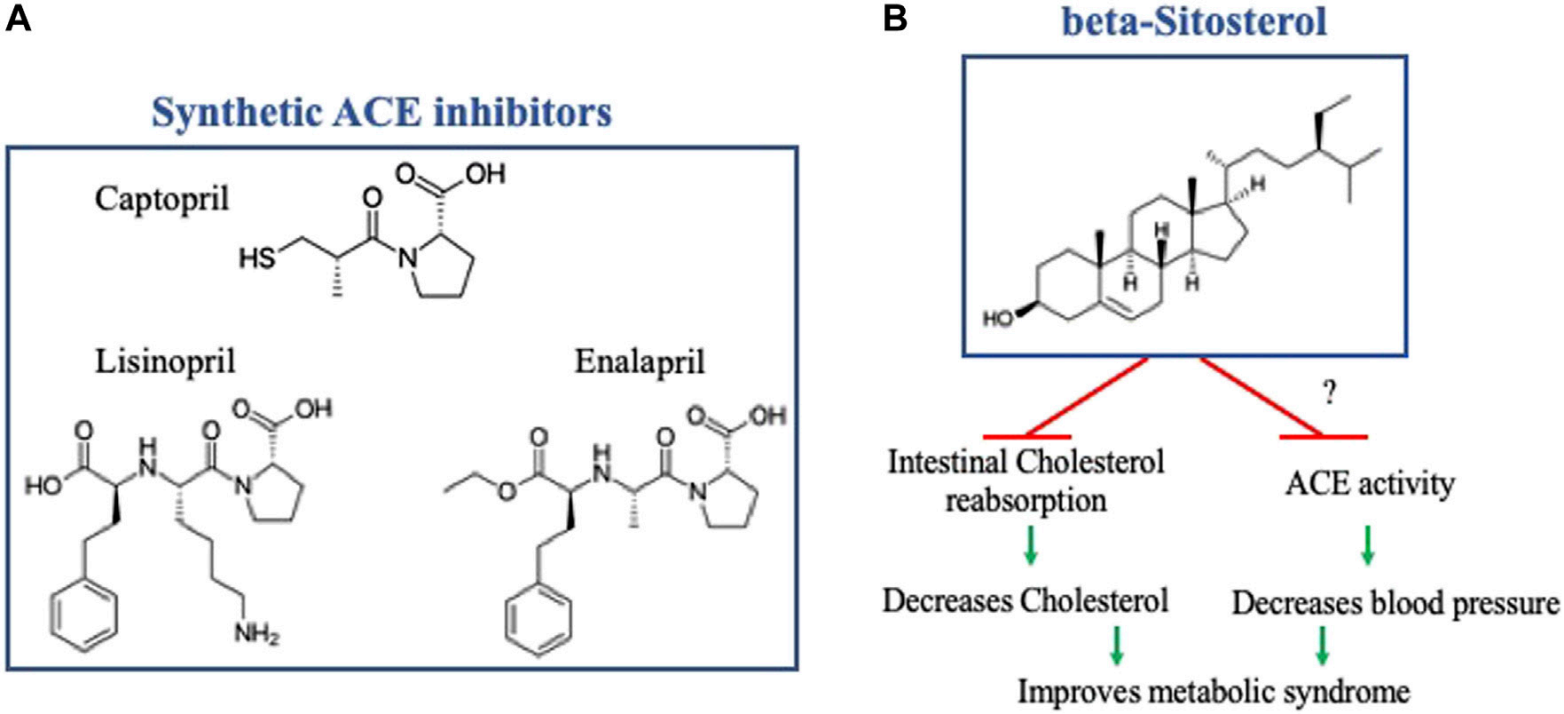
**(A)** Molecular structure of Captopril and two of its derivatives. **(B)** Molecular structure of beta-Sitosterol, note the difference from Captopril and its derivatives, with a schematic representation of its potential effect on metabolic syndrome by independently targeting increased cholesterol and blood pressure. The effect of beta- Sitosterol on inhibiting intestinal Cholesterol reabsorption has been previously established, further in-vitro and *in-vivo* studies are required to investigate its effect of ACE activity and blood pressure. The molecular structures were reproduced from Wikipedia, the free encyclopedia.

Along the same lines, Angiotensin Converting Enzyme inhibitors (ACEi), a conventional antihypertensive treatment, are also risk factors for hyperkalemia. ACEi is a class of drug regrouping synthetic molecules such as Captopril, the first approved oral ACEi, and its derivatives. The risk of hyperkalemia caused by ACEi is relatively similar among different ACEi because it directly results from their mechanism of action that reduces Aldosterone secretion, leading to potassium retention. Regardless of hyperkalemia, different ACEi molecules might differ in their lowering blood pressure efficacy and their effects on cardiac and renal function (Sun et al., 2016). Therefore, a constant search for safer ACEi with high efficacy is required. In this Research Topic, Ramlal et al., using molecular docking and dynamic simulation approaches, have analyzed six soybean bioactive compounds for their potential inhibitory effect on ACE catalytic activity. They identified beta-Sitosterol as a potential ACEi with a molecular structure different from the traditional Captopril and its derivatives (see Figure 1). Beta-Sitosterol is classified as unsaturated plant Sterol with a known Cholesterol-lowering effect [For review, see (Moghadasian and Frohlich, 1999)]. In this Research Topic, Ramlal et al. revealed an additional property of beta-Sitosterol as a potential ACEi, rendering it a potential attractive therapy for metabolic syndrome by independently targeting increased Cholesterol and blood pressure (see Figure 1). The Cholesterol-lowering effect of beta-Sitosterol has been attributed to competitive inhibition to its intestinal reabsorption due to high similarity in the molecular structures of Cholesterol and beta-Sitosterol (Moghadasian and Frohlich, 1999). Further *in vitro* and *in vivo* studies are required to investigate beta-Sitosterol effect on ACE activity and blood pressure, as well as on cardiac and renal function.

In the kidney, K^+^ secretion takes place in the distal nephron and is mediated by K^+^-secreting channels of small and big conductance (ROMK and Big-K or BK channels, respectively). ROMK and BK are exquisitely regulated by dietary K^+^ intake, a regulation that also involves the potassium counteranion (Al-Qusairi et al., 2023). In this Research Topic, the signaling pathway regulating BK channels was studied by Bi et al., who showed that SPS1-related proline/alanine-rich kinase (SPAK) is essential in regulating BK protein levels. The authors found that the knockdown of SPAK decreased, whereas SPAK overexpression increased BK protein abundance. The stimulatory effect of SPAK requires inhibiting BK lysosomal degradation, mediated by ERK1/2 signaling. They showed that SPAK-KO mice exhibit decreased BK channel abundance and a total absence of BK current in the cortical CD’s principal cells (PCs). Their study expands a previously suggested model in which WNK1 activates BK by inhibiting its ERK1/2-mediated lysosomal degradation (Liu et al., 2015). The authors suggested high potassium intake activates SPAK phosphorylation in the PCs, which would be the opposite of SPAK regulation in the DCT (Terker et al., 2015). Although cell-specific regulation of the cellular pathways is plausible, the activation of SPAK by high K^+^ in PCs needs further investigation. Interestingly, RNA-Seq analysis of mouse kidneys showed that the SPAK gene is highly expressed in PCs, even more abundantly than in the DCT (Ransick et al., 2019), suggesting it plays a crucial role in PC biology.

A U-shaped association between plasma K^+^ and All-Cause mortality, with the lowest incidence of death between (3.5 and 5 mM) (Goyal et al., 2012; Palaka et al., 2020), indicates the importance of the tight control of plasma K^+^ and the urgent need to expand our understanding of the underlying mechanisms to achieve efficient therapy. The editors are grateful to the contributors of this Research Topic who, by providing new insights into the renal regulation of K^+^ homeostasis, help enrich this field of research and open new possibilities for further investigations.
